# Migration, attrition and internal validity in longitudinal study of chronic kidney disease of unknown aetiology in Nicaragua

**DOI:** 10.1136/bmjopen-2026-122893

**Published:** 2026-11-06

**Authors:** Aurora Aragón, Indiana López-Bonilla, Neil Pearce, Dorothea Nitsch, David Gimeno Ruiz de Porras, Ben Caplin, Marvin González-Quiroz

**Affiliations:** 1 Salud y Trabajo en América Central. SALTRA Nicaragua, León, León, Nicaragua; 2 Maya Health Alliance Wuqu’ Kawoq, Tecpán Guatemala, Chimaltenango, Guatemala; 3 Department of Medical Statistics, London School of Hygiene and Tropical Medicine, London, UK; 4 Department of Non-Communicable Disease Epidemiology, London School of Hygiene and Tropical Medicine, London, UK; 5 Department of Environmental and Occupational Health, Kate Marmion School of Public Health, The University of Texas at San Antonio, San Antonio, Texas, USA; 6 Iberoamerican Social Security Organization, Madrid, Spain; 7 Hospital del Mar Medical Research Institute/Parc de Salut MAR, Barcelona, Spain; 8 Biomedical Research Networking Center for Epidemiology and Public Health, Madrid, Spain; 9 Bladder and Kidney Centre, University College London, Medical School, London, UK

**Keywords:** EPIDEMIOLOGIC STUDIES, Occupational Health, Nephrology, Adult nephrology

## Abstract

**Abstract:**

**Objectives:**

To quantify the magnitude and timing of permanent migration across three consecutive cohorts, and to assess whether migration was associated with short-term changes in kidney function in a longitudinal cohort of young adults at risk of chronic kidney disease of unknown aetiology (CKDu).

**Design:**

Community-based prospective longitudinal cohort study with repeated measurements of kidney function.

**Setting:**

Eleven rural communities in northwest Nicaragua, a region affected by CKDu.

**Participants:**

A total of 1001 young adults aged 18–30 years were recruited in three recruitment cohorts in 2014, 2018 and 2021 and followed through 2023.

**Outcome measures:**

Permanent migration was defined as confirmed departure from the study area without subsequent return during the study period.

**Results:**

Permanent migration occurred in 270 (27%) with a staggered increase. During the first 24 months, 11.2% of participants permanently migrated, corresponding to a migration rate of 6.1 per 100 person-years (95% CI 5.0 to 7.3). Migration rates were higher among men than women. Male sex was associated with a higher hazard of permanent migration both within 24 months (adjusted hazard ratio (adjHR) 2.4, 95% CI 1.5 to 3.6) and over the complete follow-up period (adjHR 2.0, 95% CI 1.5 to 2.7). Baseline estimated glomerular filtration rate (eGFR) was similar between migrants and non-migrants. Permanent migration was not associated with change in eGFR (ΔeGFR) in cohort 1 or 2 in multiple-imputation analyses. In cohort 3, migration was associated with a greater increase in eGFR (β=7.0 mL/min/1.73 m^2^, 95% CI 0.2 to 13.8), although this association was not statistically significant in the complete-case analysis (β=6.3, 95% CI −1.1 to 13.7). Male sex was associated with lower ΔeGFR across all three cohorts.

**Conclusions:**

Permanent migration was common and increased across successive recruitment cohorts, particularly among men. We found no consistent evidence that migration was associated with short-term change in eGFR. In cohort 3, estimates from the multiple-imputation and complete-case analyses were similar in magnitude and direction, although the complete-case estimate was less precise and included the null. These findings suggest that, although migration represents an important source of loss to follow-up, there was limited evidence that it was strongly associated with early kidney function change in this cohort.

**STRENGTHS AND LIMITATIONS OF THIS STUDY**

- This study directly evaluated permanent migration as a potential source of selective loss to follow-up in a long-running community-based chronic kidney disease of unknown aetiology (CKDu) cohort, an important methodological issue for longitudinal research in highly mobile rural populations.
- Migration was characterised using both time-to-event methods and person-time incidence rates, allowing assessment of both the magnitude and timing of migration across participant subgroups and recruitment cohorts.
- Repeated measurements of estimated glomerular filtration rate across three recruitment cohorts strengthened the evaluation of short-term kidney function trajectories in a highly mobile population.
- The use of a prespecified 24-month analytical window allowed more comparable assessment of early migration across cohorts with substantially different potential follow-up durations.
- Residual confounding by unmeasured social, occupational or health-related factors influencing both migration and kidney outcomes cannot be excluded.

## Introduction

 Chronic kidney disease of unknown aetiology (CKDu) has emerged as an important public health concern in agricultural communities in Mesoamerica and other low- and middle-income countries (LMICs).^1–3^ Unlike conventional chronic kidney disease (CKD) which is often associated with diabetes and hypertension, CKDu disproportionately affects working-age adults and has been associated with strenuous physical labour, heat stress, nephrotoxic medications and other occupational and environmental factors.^4 5^ Prospective community-based or occupational cohorts are particularly valuable for understanding CKDu because repeated assessment of kidney function allows identification of early changes in estimated glomerular filtration rate (eGFR), characterisation of individual kidney function trajectories and evaluation of factors associated with progression.^5–7^

A major challenge for longitudinal studies in rural LMIC populations is the mobility of working-age adults. Labour migration may be seasonal or permanent and may be driven by employment opportunities, household economic needs, agricultural cycles or broader socioeconomic conditions.^8^ In Nicaragua, internal and international migration is common among working-age populations, including workers employed in agriculture, construction, domestic work and other occupations characterised by temporary employment and geographic mobility.^8^ For longitudinal cohort studies, migration may therefore have consequences beyond the practical difficulties of participant retention. When participants leave the study area and can no longer be assessed, migration can lead to loss to follow-up and potentially reduce both the precision and validity of estimates of longitudinal change.^9^

The implication of migration-related loss to follow-up depends on whether migration occurs randomly with respect to characteristics related to the study outcomes. If participants who migrate differ systematically from those who remain-for example, in age, sex, occupation, socioeconomic circumstances, baseline kidney function or other health characteristics-analyses restricted to participants who remain under observation may produce biased estimates. This concern may be particularly relevant in CKDu research because the relationship between kidney function and migration may operate in both directions. Occupational and socioeconomic circumstances may influence both kidney health and the decision or need to migrate, while declining kidney function may itself affect physical work capacity, employment opportunities and subsequent mobility. Migration may therefore be related to the underlying kidney function trajectory, raising the possibility of informative loss to follow-up in longitudinal analyses.^6 10^

Understanding the timing and characteristics of migration is consequently important when interpreting kidney function trajectories in CKDu cohorts. A simple comparison of the proportion of participants who migrate may conceal differences in the timing of migration across demographic groups or enrolment periods. Time-to-event approaches can provide a more informative description of when migration occurs and whether the risk of migration differs according to participant characteristics. Moreover, examining kidney function change in relation to subsequent migration can help assess whether participants who leave the study differ systematically in their trajectories from those who remain under observation.

The cohort examined in this study was established in northwest Nicaragua in 2014 and initially enrolled predominantly young male agricultural workers, reflecting the prevailing recognition of CKDu as a condition primarily affecting men engaged in strenuous agricultural work.^5^ As evidence accumulated regarding CKDu among women, subsequent enrolment phases in 2018 and 2021 broadened the study population to include a greater proportion of women and provide an opportunity to examine migration patterns across sex, demographic groups and calendar periods.^11^ The longitudinal design, repeated assessment of kidney function and extended follow-up of this cohort provide a unique opportunity to examine migration not only as a challenge to participant retention but also as a potential source of selection in studies of kidney function change.

The objectives of this study were (1) to characterise the magnitude and timing of permanent migration across enrolment phases and participant subgroups and (2) to assess whether permanent migration was associated with short-term changes in kidney function, and therefore whether migration-related attrition could bias longitudinal kidney function estimates in this CKDu cohort.

## Methods

### Study design and population

We conducted a prospective community-based cohort study in 11 agricultural communities affected by CKDu along the Pacific coast of northwestern Nicaragua. The population of this region is Spanish-speaking and is largely identified as Hispanic/Latino in terms of cultural background and origin. The study was initiated in 2014 (cohort 1) and subsequently expanded through two staggered recruitment cohorts in 2018 (cohort 2) and 2021 (cohort 3), allowing for the enrolment of new participants while continuing follow-up of previously enrolled participants. Eligible individuals were aged 18–30 years at enrolment and provided written informed consent.

Recruitment in cohort 1 prioritised male farmworkers, reflecting the epidemiological evidence available at the time, which indicated a higher prevalence of CKDu among young adult men in affected communities.^12^ Recruitment targeted young adults without known CKD, diabetes or hypertension, with a planned male to female ratio of 3:1. However, some participants who appeared clinically healthy at enrolment nevertheless had reduced eGFR and were retained in the cohort for longitudinal follow-up.^1 5^ As cases of CKDu among women became increasingly recognised during the first follow-up period, subsequent recruitment cohorts intentionally increased female enrolment, resulting in a more balanced sex distribution. Details on participant enrolment, field procedures, questionnaire administration, biological sample collection, laboratory methods and community engagement have been described elsewhere.^1 5 11^

### Definition of permanent migration and monitoring

Permanent migration was defined as the absence of scheduled study visits without return after follow-up, corroborated by family members, community informants or field records. Temporary migration, defined as leaving the community with subsequent return, was not classified as an event, and participants who temporarily left the community and later returned remained under follow-up and continued contributing person-time after return. The 1001 enrolled participants were considered at risk of permanent migration from enrolment, regardless of the number of kidney function measurements available. Participants who discontinued follow-up for reasons other than permanent migration (eg, refusal, imprisonment, non-migration-related illness or death) were censored at the last confirmed contact.

### Analytical risk window

Because cohorts 1–3 had maximum potential follow-up of approximately 9, 5 and 2 years, respectively, analyses over the complete follow-up period would inherently provide a greater opportunity for migration in the earlier cohorts. We therefore predefined the first 24 months after enrolment as the primary comparison window during which all three cohorts were eligible to contribute the full observational period.

### Migration analysis

The primary analysis considered permanent migration during the first 24 months after cohort entry. This fixed analytical window was prespecified because all three cohorts had at least 24 months of potential follow-up and because maximum follow-up differed substantially between cohorts. We estimated the cumulative incidence of permanent migration using time-to-event methods, with time since cohort entry as the underlying time scale (Kaplan-Meier methods). Participants were followed from enrolment until permanent migration, censoring or completion of the 24-month window. Participants who left the study area for reasons other than permanent migration were censored at their last confirmed contact. Permanent migration rates were calculated as events per 100 person-years (PY), with 95% confidence intervals (CIs) based on exact Poisson methods. Estimates were reported both overall and stratified by sex, age group and recruitment cohort.

The same analyses were also repeated over the complete available follow-up period to describe long-term migration patterns (entire follow-up period). We used multivariable Cox proportional hazards regression to examine factors associated with time to permanent migration. Models were adjusted for baseline age group and sex, with recruitment cohort included as a stratification variable in pooled analyses. Separate models were also fitted within each cohort. Results are presented as HRs with 95% CIs. Because death precludes subsequent migration, deaths were considered competing events in a sensitivity analysis. The primary Cox analysis used cause-specific hazard, with non-migration losses to follow-up treated as censoring.

To examine whether permanent migration was associated with short-term changes in kidney function, robust linear regression models were fitted separately for each cohort, with the change in eGFR (ΔeGFR) as the outcome. Models were adjusted for sex, age group, change in body mass index (BMI) and time at risk in years.

### Kidney function analysis

Kidney function was assessed at each study visit using the Chronic Kidney Disease Epidemiology Collaboration (CKD-EPI) 2021 equation,^13^ which eGFR does not incorporate a race coefficient. The primary analysis of short-term changes in kidney function based on multiple imputation included all 1001 participants; the complete-case sensitivity analysis was restricted to participants with at least two eGFR measurements. Participants with only one eGFR measure remained eligible for migration analyses.

### Assessment of potential selection bias due to migration

To assess whether permanent migration could have introduced selection bias in estimates of renal function, we compared baseline demographic, occupational and eGFR characteristics between participants who permanently migrated and those who remained under follow-up. We then fitted robust linear regression models separately for each cohort, with short-term ΔeGFR as the outcome and permanent migration status as the main exposure. Models were adjusted for sex, age group, change in BMI (ΔBMI) and time at risk. Because participants who migrated could not be recontacted for final measurements, missing follow-up eGFR and BMI values were addressed using multiple imputation as the primary approach, with complete-case analyses conducted as a sensitivity analysis.

This analysis was used to assess whether participants who permanently migrated had different short-term kidney function trajectories from those who remained under follow-up.

### Missing data

Baseline characteristics were summarised with denominators reported for each variable. Participants with only one eGFR measurement were excluded from the complete-case sensitivity analysis of eGFR change. Missing covariate data were handled using complete-case analyses or indicator categories, as appropriate to the variable and extent of missingness. All participants had complete baseline measurements of age, sex, BMI and eGFR, as these variables were collected at enrolment before migration occurred. Missing follow-up BMI and eGFR measurements arose among participants who permanently migrated and could not be recontacted or did not return to their community of origin for a follow-up assessment. Because missing follow-up measurements were associated with observed migration status and sex, multiple imputation was performed under a missing-at-random (MAR) assumption conditional on these and other observed covariates. This assumption cannot be verified from the observed data. Missing follow-up eGFR and BMI values were therefore addressed using multiple imputation by chained equations as the primary approach.

Thirty imputed datasets were generated using predictive mean matching. The imputation model included baseline eGFR, baseline BMI, sex, age group, time at risk and permanent migration status. Regression estimates were calculated separately within each imputed dataset and combined using Rubin’s rules. A complete-case analysis was conducted as a sensitivity analysis. Because the missing follow-up measurements were concentrated among participants who permanently migrated, the MAR assumption cannot be verified from the observed data. The complete-case analysis was therefore used to assess the robustness of the findings to the handling of missing follow-up measurements.

## Results

### Cohort characteristics

A total of 1001 participants were enrolled across three cohorts in agricultural communities along the Pacific coast of northwestern Nicaragua. Baseline characteristics differed across recruitment cohorts, particularly with respect to age, sex and occupation. All participants were identified as Hispanic/Latino. Participants in cohort 3 were younger than those in cohort 1, with 68.4% aged 18–22 years compared with 42.9% in cohort 1. The proportion of women increased from 24.6% in cohort 1 to approximately 50% in cohorts 2 and 3. Daily income distribution and BMI were broadly similar across cohorts, whereas employment patterns changed over time. Agricultural work was more common in cohort 1, while non-agricultural and domestic work became more prevalent in later cohorts, accompanied by a decline in unemployment. Mean baseline eGFR was above 100 mL/min/1.73 m^2^ in all three cohorts. Follow-up duration was longer in the earlier cohorts, reflecting the staggered recruitment (table 1).

**Table 1:** Baseline (cohort entry) characteristics (n and %*) of participants by cohort

Characteristics†	Cohort 1 (n=350)	Cohort 2 (n=420)	Cohort 3 (n=231)	Total (n=1001)
Age at baseline, in years (mean, SD)	23.5 (3.7)	23.0 (3.6)	21.6 (3.2)	22.9 (3.6)
Age group**,** in years (n, %)				
18–22	150 (42.9)	207 (49.3)	158 (68.4)	515 (51.4)
23–30	200 (57.1)	213 (50.7)	73 (31.6)	486 (48.6)
Sex (n, %)				
Male	264 (75.4)	214 (51.0)	120 (51.9)	598 (59.7)
Female	86 (24.6)	206 (49.0)	111 (48.1)	403 (40.3)
Daily income, in US$ (n, %)‡				
0.17–2.50	203 (62.7)	262 (62.4)	141 (61.0)	606 (62.2)
2.51–16.38	121 (37.3)	158 (37.6)	90 (39.0)	369 (37.8)
Body mass index (mean, SD)	23.9 (4.7)	24.5 (4.6)	24.4 (4.7)	24.2 (4.6)
Employment (n,%)§				
Agricultural work (eg, sugarcane, banana)	168 (48.0)	131 (31.3)	63 (27.3)	362 (36.2)
Non-agricultural work (eg, construction, trade)	76 (21.7)	139 (33.2)	88 (38.1)	303 (30.3)
Domestic/home-based work	55 (15.7)	126 (30.1)	69 (29.9)	250 (25.0)
Unemployed (able or unable to work)	51 (14.6)	23 (5.5)	11 (4.8)	85 (8.5)
Baseline eGFR in mL/min/1.73 m^2^				
Mean (SD)	124.8 (20.3)	117.3 (20.6)	110.9 (20.0)	118.5 (21.0)
Minimum–maximum	29.4–158.2	28.4–170.9	30.4–158.2	28.4–170.9
Follow-up time (in years)				
Mean (SD)	7.0 (2.5)	4.1 (1.3)	1.6 (0.6)	4.5 (2.7)
Minimum–maximum	0.3–9.0	0.5–5.0	0.5–2	0.3–9.0

* Column percentages.

† All participants were Hispanic/Latino and were recruited from the same geographic region.

‡ 26 participants refused.

§ One participant refused.

eGFR, estimated glomerular filtration rate.

### Permanent migration during follow-up

During the first 24 months of follow-up, 112 participants (11.2%) permanently migrated, corresponding to an overall migration rate of 6.1 per 100 PY (95% CI 5.0 to 7.3) (table 2, panel A). Migration rates were higher among men than women (7.5 vs 3.9 per 100 PY) and among participants aged 18–22 years compared with those aged 23–30 years (7.1 vs 5.0 per 100 PY). Migration rates also increased substantially across recruitment cohorts, from 3.1 per 100 PY in cohort 1 to 13.6 per 100 PY in cohort 3 (table 2, panel A). Most non-migratory loss to follow-up (75.3%) was due to temporary work-related absences without confirmed permanent migration. Refusal to continue participation and death due to CKD each accounted for 6.5% of these losses (online supplemental table S2).

**Table 2:** Cumulative incidence and incidence rates of permanent migration by cohort, age and sex

Cohorts	Characteristics		Baseline (n)	Permanent migrants (n)	% Cumulative incidence (95% CI)	Person-years (PY) at risk^*^	Incidence rate/100 PY (95% CI)	Crude rate ratio (95% CI)	Crude HR (95% CI)	Adjusted† HR (95% CI)
**A. Within 24 months of follow-up**										
All cohorts**	Age (years)**^‡^**	18–22	515	66	12.8 (9.8 to 15.8)	933	7.1 (5.5 to 9.0)	1.4 (0.9 to 2.0)	1.4 (0.9 to 2.0)	1.0 (0.7 to 1.5)
		23–30	486	46	9.5 (6.8 to 12.2)	912.3	5.0 (3.7 to 6.7)	Ref		
	Sex	Male	598	82	13.7 (10.9 to 16.6)	1090.3	7.5 (5.9 to 9.3)	1.9 (1.2 to 2.9)	1.9 (1.3 to 2.9)	2.4 (1.5 to 3.6)
		Female	403	30	7.4 (4.8 to 10.1)	755	3.9 (2.7 to 5.7)	Ref		
	Cohort	1	350	21	6.0 (3.4 to 8.6)	680.3	3.1 (1.9 to 4.7)	Ref		
		2	420	42	10.0 (7.0 to 12.9)	805.5	5.2 (3.8 to 7.0)	1.7 (1.0 to 3.0)		
		3	231	49	21.2 (15.7 to 26.7)	359.5	13.6 (10.1 to 18.0)	4.4 (2.6 to 7.8)		
	Overall		1001	112	11.2 (9.2 to 13.2)	1845.3	6.1 (5.0 to 7.3)			
**B. Over the complete follow-up period**										
All cohorts	Age (years)**^‡^**	18–22	515	150	29.1 (25.1 to 33.1)	2084.3	7.2 (6.1 to 8.4)	1.5 (1.2 to 1.9)	1.5 (1.1 to 1.9)	1.2 (0.9 to 1.6)
		23–30	486	120	24.7 (20.8 to 28.6)	2444.3	4.9 (4.1 to 5.9)	Ref.		
	Sex	Male	598	195	32.6 (28.5 to 36.5)	2768	7.0 (6.0 to 8.1)	1.7 (1.3 to 2.2)	1.7 (1.3 to 2.2)	2.0 (1.5 to 2.7)
		Female	403	75	18.6 (14.7 to 22.5)	1760.5	4.2 (3.3 to 5.3)	Ref.		
	Overall	* *	1001	270	26.9 (24.2 to 29.8)	4528.5	5.9 (5.2 to 6.7)			
Cohort 1 (2014)	Age (years)**^‡^**	18–22	150	51	34.0 (26.1 to 41.9)	1021.3	5.0 (3.7 to 6.6)	1.3 (0.9 to 1.9)	1.3 (0.9 to 2.0)	1.3 (0.9 to 1.9)
		23–30	200	53	26.5 (20.1 to 32.9)	1428.3	3.7 (2.8 to 4.9)	Ref.		
	Sex	Male	264	82	31.1 (25.3 to 36.8)	1791.5	4.6 (3.6 to 5.7)	1.4 (0.9 to 2.2)	1.4 (0.9 to 2.2)	1.4 (0.9 to 2.2)
		Female	86	22	25.6 (15.8 to 35.4)	658.0	3.3 (2.1 to 5.1)	Ref.		
	Overall		350	104	29.7 (24.8 to 34.6)	2449.5	4.2 (3.4 to 5.1)			
Cohort 2 (2018)	Age (years)**^‡^**	18–22	207	64	30.9 (24.4 to 37.5)	818.0	7.8 (6.0 to 9.9)	1.3 (0.9 to 1.9)	1.4 (0.9 to 1.9)	1.2 (0.8 to 1.8)
		23–30	213	53	24.9 (18.8 to 30.9)	901.5	5.8 (4.4 to 7.7)	Ref.		
	Sex	Male	214	80	37.4 (30.7 to 44.1)	802.5	9.9 (7.9 to 12.4)	2.5 (1.7 to 3.7)	2.6 (1.7 to 3.8)	2.5 (1.7 to 3.7)
		Female	206	37	17.9 (12.5 to 23.4)	917.0	4.0 (2.8 to 5.6)	Ref.		
	Overall		420	117	27.9 (23.5 to 32.3)	1719.5	6.8 (5.6 to 8.2)			
Cohort 3 (2021)	Age (years)**^‡^**	18–22	158	35	22.2 (15.4 to 28.9)	245.0	14.3 (9.9 to 19.9)	1.2 (0.6 to 2.2)	1.1 (0.6 to 2.2)	1.1 (0.6 to 2.0)
		23–30	73	14	19.2 (9.5 to 28.9)	114.5	12.3 (6.71 to 20.0)	Ref		
	Sex	Male	120	33	27.5 (19.1 to 35.9)	174.0	18.9 (13.05 to 26.6)	2.2 (1.2 to 3.9)	2.1 (1.2 to 3.9)	2.1 (1.2 to 3.9)
		Female	111	16	14.4 (7.4 to 21.4)	185.5	8.7 (4.9 to 14.0)	Ref		
	Overall		231	49	21.2 (15.7 to 26.7)	359.5	13.6 (10.1 to 18.0)			

* Person-time accrued until migration, *censoring *or *end of follow-up*

† HRs were adjusted for baseline age group and sex. For “All cohorts”, cohort was included as a stratification varaible. Separate models were fitted within each cohort.

‡ Participant age at baseline

Within each cohort, differences in migration rates by age group were not statistically significant. Sex differences were statistically significant in cohorts 2 and 3. Analyses over the complete follow-up period showed similar patterns, although based on a larger period of observation (table 2, panel B). Migration trajectories by sex and recruitment cohort are illustrated in online supplemental figure S1.

### Predictors of permanent migration

In the multivariable Cox proportional hazards models, male sex was consistently associated with a higher hazard of permanent migration. Within the first 24 months, men had more than twice the hazard of migration compared with women (adjHR 2.4, 95% CI 1.5 to 3.6). This association remained evident over the complete follow-up period (adjHR 2.0, 95% CI 1.5 to 2.7). The association with male sex was similar in magnitude across cohorts, although the estimate for cohort 1 did not reach statistical significance (cohort 1: HR 1.4, 95% CI 0.9 to 2.2; cohort 2: HR 2.5, 95% CI 1.7 to 3.7; cohort 3: HR 2.1, 95% CI 1.2 to 3.9). Age group was not a statistically significant predictor of permanent migration in the adjusted models (table 2).

### Baseline estimated glomerular filtration rate by cohort and permanent migration status

Median baseline eGFR was similar among participants with and without permanent migration, both overall (124.3 vs 123.4 mL/min/1.73 m^2^; p=0.538) with median values exceeding 100 mL/min/1.73 m^2^ in both groups within each cohort (table 3). Overall, 21 participants (2.1%) had a baseline eGFR <60 mL/min/1.73 m^2^: 5 of 270 participants with permanent migration (1.9%) and 16 of 731 participants without permanent migration (2.2%; Fisher’s exact p=1.000; online supplemental table S1).

**Table 3:** Comparison of baseline eGFR (median, IQR) between participants with and without permanent migration, by cohort

Cohorts	Permanent migration		P value*
	Yes	No	
	Baseline eGFR in mL/min/1.73 m^2^ Median (P25–P75)	Baseline eGFR in mL/min/1.73 m^2^ Median (P25–P75)	
All cohorts	124.3 (107.9–130.4)	123.4 (106.5–130.7)	0.538
Cohort 1 (2014)	128.7 (121.6–137.8)	126.9 (119.9–135.9)	0.329
Cohort 2 (2018)	121.7 (104.9–127.7)	123.4 (105.7–130.4)	0.139
Cohort 3 (2021)	120.8 (104.9–126.0)	109.4 (100.6–125.1)	0.223

* P value of Mann-Whitney U test.

eGFR, estimated glomerular filtration rate.

### Missing follow-up measurements

Follow-up eGFR and BMI were missing for 52 participants (5.2%), all of whom had permanently migrated. Missingness was higher among men than women (6.4% vs 3.5%; p=0.044). All missing measurements occurred among participants who had permanently migrated and could not be located or did not return to their community of origin for follow-up assessment.

### Association between permanent migration and change in estimated glomerular filtration rate

In the primary multiple-imputation analyses, permanent migration was not associated with short-term change in eGFR in cohort 1 (β=2.8 mL/min/1.73 m^2^, 95% CI −4.7 to 10.3) or cohort 2 (β=2.6, 95% CI −4.4 to 9.6). In cohort 3, permanent migration was associated with a greater increase in eGFR (β=7.0 mL/min/1.73 m^2^, 95% CI 0.2 to 13.8) (table 4). However, this association was not statistically significant in the complete-case sensitivity analysis (β=6.3, 95% CI −1.1 to 13.7) (Online supplemental table S3). Male sex was consistently associated with lower ΔeGFR across all three cohorts, with β estimates of −15.7 mL/min/1.73 m^2^ (95% CI −20.9 to −10.6) in cohort 1, −20.4 (95% CI −25.0 to −15.9) in cohort 2 and −14.8 (95% CI −19.5 to −10.0) in cohort 3. Baseline age was not associated with ΔeGFR in any cohort. In cohort 3, longer time at risk was associated with a greater increase in eGFR (table 4).

**Table 4:** Multivariable robust regression models of change in eGFR across the three cohorts using multiply imputed data under the MAR assumption (n=1001)

Characteristics	Cohort 1 (n=350) β (95% CI)	Cohort 2 (n=420) β (95% CI)	Cohort 3 (n=231) β (95% CI)
Permanent migration (yes)	2.8 (−4.7 to 10.3)	2.6 (−4.4 to 9.6)	7.0 (0.2 to 13.8)
Sex (male)	−15.7 (−20.9 to −10.6)	−20.4 (−25.0 to −15.9)	−14.8 (−19.5 to −10.0)
Age (18–22)	1.0 (−4.0 to 6.0)	−2.5 (−6.3 to 1.3)	2.1 (−3.2 to 7.4)
BMI difference	0.6 (−0.3 to 1.4)	−0.2 (−0.9 to 0.5)	0.9 (−0.1 to 1.8)
Time at risk (years)	−0.7 (−2.2 to 0.9)	−0.6 (−3.0 to 1.7)	4.9 (0.8 to 9.1)
R^2^	0.09	0.206	0.219
Missing data	3 (0.9%)	13 (3.1%)	36 (15.6%)

From robust linear regression models with multiple imputation (MICE; 30 imputed datasets) for missing follow-up eGFR/BMI under MAR, pooled using Rubin’s rules. R2 values for the 2014, 2018 and 2021 cohorts were 0.090, 0.206, and 0.219, respectively

BMI, body mass index; eGFR, estimated glomerular filtration rate; MAR, missing-at-random; MICE, multiple imputation by chained equations.

## Discussion

This study examined whether permanent migration constituted a source of selective attrition in a long-standing CKDu cohort in northwest Nicaragua. Permanent migration occurred among 11.2% of participants within the first 24 months after enrolment and among 27.0% over the complete follow-up period, with higher migration among men and descriptively higher migration rates among younger adults. Nevertheless, migrants and non-migrants had similar baseline kidney function, and we found no consistent evidence that permanent migration was associated with short-term change in eGFR. In cohort 3, permanent migration was associated with a greater increase in eGFR in the primary multiple-imputation analysis. However, the corresponding estimate from the complete-case analysis was similar in magnitude and direction but less precise, with a 95% CI that included the null. Thus, the apparent difference between the analyses reflected statistical uncertainty rather than contradictory effect estimates. Overall, permanent migration represented an important source of loss to follow-up, but there was little evidence that it was strongly associated with early kidney function trajectories. Nevertheless, informative censoring cannot be excluded. Migration rates and cumulative migration proportions both increased across recruitment cohorts, indicating more rapid early exits among recently enrolled participants.

The observed migration patterns are consistent with broader labour dynamics in rural Nicaragua, where seasonal employment, informal work arrangements and economic insecurity contribute to high mobility among younger workers, particularly men.^8 14^ In this context, migration may reflect livelihood strategies rather than health-related selection. This distinction is important because participant loss in cohort studies is often assumed to threaten validity;^15^ however, the degree of bias depends not only on the amount of attrition but also on whether those lost differ systematically from those retained.^16^ The higher migration observed in later cohorts may reflect changes in the economic conditions and labour context during the study period, although the present study did not directly measure the mechanisms underlying these temporal differences.^14 17 18^

An additional consideration is that participants with clinically significant kidney function decline were not simply observed passively during follow-up. Participants with abnormal kidney function measurements were informed of their results and referred for clinical evaluation when appropriate either through the Ministry of Health or the Social Security System. When necessary, participants were also supported in accessing haemodialysis programmes. This active follow-up may have influenced retention among participants with recognised kidney dysfunction; however, the extent to which clinical referral affected subsequent migration or study participation could not be quantified.^1 5 11^ Although these actions do not eliminate the possibility of selection processes during follow-up, they may have reduced the likelihood that severely affected participants disengaged from the cohort without clinical recognition or referral. This active clinical feedback loop may therefore have mitigated one potential pathway of informative censoring related to disease severity.^19^

Few CKDu studies have formally evaluated migration as a potential source of informative censoring in relation to declining kidney function.^20 21^ Prior work suggests that labour mobility among rural agricultural populations is often shaped by broader economic and environmental pressures, including climate-related stressors, which may complicate retention in longitudinal research.^17^ Our findings extend this literature by quantifying migration using both cumulative incidence and person-time metrics, and by explicitly examining whether migration was associated with kidney function trajectories. The results indicate that substantial attrition does not necessarily imply major analytic bias.^9^

Although migration was not strongly associated with kidney function decline, male participants consistently showed steeper reductions in eGFR across recruitment cohorts. This pattern is consistent with previous studies from Nicaragua and the wider Mesoamerican region and may reflect persistent differences in occupational heat exposure, workload intensity, hydration practices or other sex-linked vulnerabilities.^22^ The combination of higher mobility and faster decline among men underscores the need to separately examine structural mobility from disease-related selection.

This study has several strengths. It is the first and longest prospective cohort study of CKDu to include multiple enrolment periods spanning nearly a decade. This longitudinal design enabled us to assess CKDu incidence over an extended period and compare migration rates across cohorts recruited at different time points. Permanent migration was defined using community verification and field records, helping distinguish permanent exits from temporary mobility. Use of a pre-specified 24-month analytical window improved comparability across cohorts with differing potential follow-up times. Furthermore, kidney function was estimated using the CKD-EPI 2021 equation, which does not incorporate a race coefficient. This approach eliminated race-based adjustment as a source of differential eGFR estimation, supporting consistent comparisons of kidney function decline across the cohorts. Finally, analyses conducted over the entire follow-up period yielded similar results, supporting the robustness of the findings.

Several limitations should also be considered. Classification of migration relied partly on family or community reports and may have misclassified some circular or temporary migration. Kidney function was measured at discrete study visits, limiting sensitivity to short-term fluctuations. Residual confounding from unmeasured socioeconomic or occupational factors remains possible. Although permanent migration was not strongly associated with kidney function decline, other forms of healthy worker selection may have occurred through job change, reduced workload, temporary mobility or continued participation among healthier individuals. Moreover, because kidney function after permanent migration was not observed, preserved kidney function before migration cannot establish that participants remained free of subsequent kidney function decline. This is particularly relevant in CKDu, which may develop without clinically apparent symptoms. Thus, informative censoring cannot be excluded despite the similarity in observed kidney function between migrants and non-migrants. In addition, our assessment focused on early follow-up; migration-related bias could emerge over longer periods if health status increasingly influences relocation or continued participation. Enrolment was intentionally restricted to individuals aged 18–30 years, given that CKDu disproportionately affects young men of working age.^12^ This approach enabled us to identify apparently healthy young adults early in their working lives and follow them prospectively over time. The three cohorts were recruited using consistent eligibility criteria from communities in the León and Chinandega coastal zone, an export-oriented agro-industrial region. However, cohort 3 was recruited during a period of regional economic instability and reduced employment opportunities. These differences in the socioeconomic context at the time of recruitment may have contributed to the higher migration observed in cohort 3, although this hypothesis cannot be established from the present data.

These findings have implications beyond Nicaragua. Many longitudinal studies in LMIC settings face high mobility driven by economic necessity. Our results suggest that rather than assuming attrition necessarily invalidates follow-up studies, investigators should empirically assess whether losses are selective and outcome related. Approaches such as inverse probability weighting, enhanced participant tracing and sensitivity analyses may be especially useful as follow-up accrues.^23^ In regions heavily affected by CKDu, where the burden on haemodialysis programmes continues to increase, periodic monitoring of kidney function should be considered an essential public health strategy, even while the underlying causes of the disease remain under investigation.

In conclusion, permanent migration represented a substantial source of loss to follow-up in this longitudinal CKDu cohort, with migration rates increasing across successive recruitment cohorts, particularly among men. Migrants had baseline kidney function like that of participants who remained under follow-up, and we found no consistent evidence that permanent migration was associated with short-term change in eGFR. The estimates for cohort 3 were similar in magnitude and direction across analyses, although the complete-case estimate was less precise and its CI included the null. These findings provide little evidence that migration was strongly associated with early kidney function trajectories, but they do not exclude informative censoring or selection arising from unmeasured social, occupational or health-related factors. Longitudinal CKDu studies in highly mobile populations should therefore monitor migration systematically and incorporate sensitivity analyses for potentially informative loss to follow-up.

## Supplementary material

10.1136/bmjopen-2026-122893 online supplemental file 1

## Data Availability

Data are available upon reasonable request.
