# Supplementary material for: Migration, attrition and internal validity in longitudinal study of chronic kidney disease of unknown aetiology in Nicaragua
Source: BMJ Open. 2026 Nov 6;16(11):e122893. doi: 10.1136/bmjopen-2026-122893 (PMC13756283; doi:10.1136/bmjopen-2026-122893)
Supplement: Supplementary file 1 [file bmjopen-2026-122893.reviewer_comments.pdf]

PEER REVIEW HISTORY

BMJ Open publishes all reviews undertaken for accepted manuscripts. Reviewers are asked to complete a checklist review form and are provided with free text boxes to elaborate on their assessment. These free text comments are reproduced below.

ARTICLE DETAILS

Title (Provisional)

Migration, Attrition, and Internal Validity in a Longitudinal Study of Chronic Kidney Disease of Unknown Aetiology in Nicaragua

Authors

Aragón, Aurora; López , Indiana; Pearce, Neil; Nitsch, Dorothea; Gimeno Ruiz de Porras, David; Caplin, Ben; Gonzalez-Quiroz, Marvin

---

VERSION 1 - REVIEW

**Reviewer**	**1**
**Name**	**Luo, Yang**
**Affiliation**	**Beijing Shijitan Hospital, Department of Nephrology**
**Date**	**26-Jul-2026**
**COI**	**None**

This is the first quantitative analysis of migration-related attrition bias in CKDu cohorts. Standardized 24-month analytical windows and clear differentiation between temporary labor mobility and permanent migration strengthen methodological rigor. The consistent finding of accelerated eGFR decline in males offers meaningful regional epidemiological insight for mobile agricultural populations. However, there are still some major concerns about this manuscript.

First, the study only enrolled participants aged 18–30 years. As the authors wanted to validate the influence of migration on the decline of kidney function, information on middle-aged individuals also needed to be presented, as we may understand, age is an established independent risk factor for CKD progression. If we have limited information among those individuals, maybe the conclusions in this study are somewhat improper.

Secondly, the authors in this study didn't compare migration rates between participants with normal and impaired baseline eGFR, which weakens the evidence regarding differential loss to follow-up by disease severity.

As the work provides valuable methodological evidence for CKD longitudinal studies in low/middle-income regions, the narrow age range substantially reduces external validity.

**Reviewer**	**2**
**Name**	**Kelson, Zoe**
**Affiliation**	**University of Exeter, Mathematics**
**Date**	**10-Aug-2026**
**COI**	**None**

This study aims to quantify the magnitude and timing of permanent migration across three consecutive cohorts, and to assess whether migration was associated with shortterm changes in kidney function, potentially threatening the internal validity of longitudinal kidney function estimates in this CKDu cohort.

Reviewer comments:

"Eleven rural communities in northwest Nicaragua, a region affected by chronic kidney disease of unknown aetiology (CKDu). Participants: A total of 1,001 young adults aged 18–30 years were recruited in three cohorts (2014, 2018, and 2021) and followed through 2023, with follow-up ranging up to nine years. " [Abstract]

and

"We conducted a prospective community-based study in 11 communities affected by chronic kidney disease in northwestern Nicaragua. The study was initiated in 2014 (Cohort 1) and subsequently expanded through two staggered recruitment cohorts in 2018 (Cohort 2) and 2021 (Cohort 3)..."

Can the authors please futher comment on whether the included cohorts can be considered to be representative?

"We estimated the cumulative incidence of permanent migration within the first 24 months using Kaplan-Meier methods, with time since cohort entry as the underlying time scale. "

Can the authors please also consider adjusting for covariates in a Cox regression model?

"To examine whether permanent migration was associated with short-term changes in kidney function, robust linear regression models were fitted separately for each cohort, with the change in estimated glomerular filtration rate (ΔeGFR) as the outcome. Models were adjusted for sex, change in body mass index (BMI), and time at risk in years. "

Appropriate modelling methods have been applied.

Can age, comorbidities, and lifestyle factors please be considered for inclusion in the models?

"We also fitted robust linear regression models with short-term change in eGFR (ΔeGFR) as the outcome and migration status as the primary exposure, adjusting for sex, change in BMI, and time at risk"

Insightful addditional analyses have been undertaken by the authors.

"Baseline characteristics were analyzed using a complete-case approach, with denominators reported for each variable. Participants with only one eGFR measurement were excluded from slope estimation by design. Missing covariate data were handled using indicator categories or complete-case modeling, depending on the extent and pattern of missingness."

Can the authors please assess whether missing data can be considered to be missing at random?

"Table 2. Baseline (cohort entry) characteristics (n and %*) of participants by cohort."

Can race and ethnicity please be reported here?

"Residual confounding by unmeasured social, occupational, or health-related factors influencing both migration and kidney outcomes cannot be excluded." [Strengths and Limitations of this study]

and

"Several limitations should also be considered. Classification of migration relied partly on family or community reports and may have misclassified some circular or temporary migration. Kidney function was measured at discrete study visits, limiting sensitivity to short-term fluctuations. Residual confounding from unmeasured socioeconomic or occupational factors remains possible. Although permanent migration was not strongly associated with kidney function decline, other forms of healthy worker selection may have occurred through job change, reduced workload, temporary mobility, or continued participation among healthier individuals. In addition, our assessment focused on early follow-up; migration-related bias could emerge over longer periods if health status increasingly influences relocation or continued participation."

The authors have provided a Discussion on the study limitations.

Thanks to the authors for providing a copy of the STROBE checklist.

VERSION 1 - AUTHOR RESPONSE

Reviewer 1 (Dr. Yang Luo)

Comment: *The age range restricted to 18-30 years at enrolment limits the external validity of the findings and their applicability to middle-aged adults.*

Response:

The restriction to participants aged 18-30 years at enrolment was a deliberate recruitment strategy designed to study individuals early in their working lives, before the onset of common age-related comorbidities, and to establish a baseline from which early declines in kidney function could be detected through annual follow-up. This approach is particularly relevant in our study because chronic kidney disease of unknown aetiology (CKDu) disproportionately affects young and early-middle-aged agricultural workers in the study region. We acknowledge, however, that the age-restricted cohort may limit the generalizability of our findings to older agricultural workers and to middle-aged adults more broadly. Accordingly, we have added a statement to the Limitations section noting that migration-related attrition and kidney functions trajectories may differ in older populations not represented in this cohort.

Comment: *The study did not compare migration rates between participants with normal versus impaired baseline eGFR, which weakens the evidence on differential loss to follow-up by disease severity.*

Response:

In response to the reviewer's concern, we have expanded the assessment of baseline kidney function according to subsequent permanent migration status. Median baseline eGFR was similar among participants with and without permanent migration overall (124.3 vs 123.4 mL/min/1.73 m²; p=0.538), with median values exceeding 100 mL/min/1.73 m² in both groups within each cohort (Table 3). Overall, only 21 participants (2.1%) had a baseline eGFR <60 mL/min/1.73 m²: 1.9% (5/270) participants with permanent migration and 2.2% (16/731) participants without permanent migration (Fisher's exact p=1.000; Supplementary Table S1).

We also fitted a multivariable Cox model for time to permanent migration, with sex and age group as covariates and cohort as a stratification variable in the pooled model, with additional cohort-specific models. Baseline eGFR was not included as a covariate because descriptive analyses showed little evidence of differences in baseline kidney function by subsequent migration status. Male sex was associated with permanent migration in both the 24-month analysis (aHR=2.4; 95%CI: 1.5-3.6) and the complete follow-up period (aHR=2.0; 95%CI: 1.5-2.7). In the cohort-specific models, the corresponding estimates were aHR=1.4; 95%CI: 0.9-2.2 in Cohort 1, aHR=2.5; 95%CI: 1.7-3.7 in Cohort 2, and aHR=2.1; 95%CI: 1.2-3.9 in Cohort 3. By contrast, age group (18-22 vs 23-30 years) was not a statistically significant predictor in the adjusted models. These results are now reported in Table 2, described in the Results, and discussed in the Discussion.

Reviewer 2 (Prof. Zoe Kelson)

Comment: *The representativeness of the three cohorts should be more clearly established.*

Response:

All three cohorts (2014, 2018, and 2021) were recruited from the same communities in the coastal zone between León and Chinandega, western Nicaragua, using the same eligibility criteria and recruitment approach. As a result, the cohorts are comparable to one another and representative of the young agricultural workforce from this CKD-endemic region that constitutes the target population of study. We acknowledge, however, that their representativeness is limited to agricultural communities with similar occupational and environmental conditions and may not be generalised to agricultural populations in other regions or age groups. We have added a clarification to the Limitations section about this representativeness issue.

Comment: *Consider adjusting for covariates via Cox regression rather than, or alongside, Kaplan-Meier.*

Response:

Please see our response to the second comment from Reviewer 1, above. Briefly, we supplemented the Kaplan-Meier analyses with multivariable Cox proportional hazards regression models for time to

permanent migration, adjusted for sex and age group and stratified by cohort. The Cox model results were consistent with the Kaplan-Meier findings and add covariate-adjusted estimates of migration risk.

Comment: *Consider adding age, comorbidities, and lifestyle factors to the ΔeGFR robust regression models (currently adjusted for sex, ΔBMI, and time at risk only).*

Response:

We added baseline age to the multivariable robust regression models for ΔeGFR (Table 4) in both the primary multiple-imputation analysis and the complete-case sensitivity analysis (Supplementary Table S3). Age was not a significant predictor of ΔeGFR in any cohort under either approach (MI model: Cohort 1, β=1.0; 95%CI: -4.0 to 5.8]; Cohort 2, β=-2.5; -95CI: 6.3 to 1.3; Cohort 3, β=2.1; 95%CI: -3.2 to 7.4). Inclusion of age did not meaningfully alter the association in Cohort 3: age-adjusted β=7.0; 95%CI: 0.2-13.8 vs. original β=7.1; 95%CI: 0.4-13.8). We did not adjust for comorbidities because the study population was intentionally restricted to apparently healthy young adults without diabetes, hypertension, or other known chronic diseases at enrollment. Moreover, neither smoking nor alcohol consumption was not associated with subsequent decline in eGFR in our previous publication, because the prevalence of these factors are lower in this population.

Comment: *Can the missing data be considered missing at random (MAR)?*

Response:

Missing follow-up eGFR and BMI measurements occurred among 52 participants (5.2% of the total sample; 0.9%, 3.1%, and 15.6% in Cohorts 1, 2, and 3, respectively), all of whom had permanently migrated and could not be recontacted or did not return to their community of origin for follow-up assessment. Missingness was also somewhat higher among men than women (6.4% vs 3.5%; p=0.044). Thus, missingness with observed characteristics (migration status and sex) rather than with missing baseline data, as all participants had complete baseline measurements of age, sex, BMI and eGFR.

We therefore considered the MAR assumption to be a reasonable working assumption conditional on observed covariates and used multiple imputations by chained equations (MICE) as our primary analytical approach. the imputation model included baseline eGFR, baseline BMI, sex, age group, time at risk and permanent migration status. However, we acknowledge that the MAR assumption cannot be verified from the observed data and that informative censoring related to unobserved post-migration health status cannot be excluded.

To evaluate the robustness of our findings, we also performed a complete-case sensitivity analysis. Results were broadly consistent with those from MICE. For example, in Cohort 3, the estimated association between permanent migration and ΔeGFR was similar in magnitude in the complete-case estimate (β=6.3; 95%CI: -1.1 to 13.7) and the MI estimate (β=7.0; 95%CI: 0.2-13.8), although the complete-case estimate was less precise.

Comment: *Please report race/ethnicity in Table 2 (baseline characteristics).*

Response:

All participants were recruited from agricultural communities in Nicaragua's Pacific/western region, resulting in a relatively homogeneous study population with respect to geographic and sociodemographic characteristics. Race and ethnicity were therefore not expected to contribute substantially to heterogeneity within the cohort, as all participants identified as Hispanic/Latino. Importantly, the 2021 CKD-EPI equation used to estimate eGFR does not incorporate a race coefficient. Thus, race-based adjustment was not a source of differential eGFR estimation within this cohort and is unlikely to have introduced bias in comparisons of eGFR decline among participants..

VERSION 2 - REVIEW

**Reviewer**	**2**
**Name**	**Kelson, Zoe**
**Affiliation**	**University of Exeter, Mathematics**
**Date**	**07-Oct-2026**
**COI**	

Thanks to the authors for responding to each comment in turn, undertaking further analyses and revising the reporting of the study accordingly.

Author response:

"All participants were recruited from agricultural communities in Nicaragua's Pacific/western region, resulting in a relatively homogeneous study population with respect to geographic and sociodemographic characteristics. Race and ethnicity were therefore not expected to contribute substantially to heterogeneity within the cohort, as all participants identified as Hispanic/Latino. Importantly, the 2021 CKD-EPI equation used to estimate eGFR does not incorporate a race coefficient. Thus, race-based adjustment was not a source of differential eGFR estimation within this cohort and is unlikely to have introduced bias in comparisons of eGFR decline among participants"

Reviewer requets:

Can this response to reviewer please be incorporated into the article, reporting race and ethnicity (if known) in the table of baseline characteristics?

VERSION 2 - AUTHOR RESPONSE

Reviewer 2 (Prof. Zoe Kelson)

Comment: *Author response:*
"All participants were recruited from agricultural communities in Nicaragua's Pacific/western region, resulting in a relatively homogeneous study population with respect to geographic and sociodemographic characteristics. Race and ethnicity were therefore not expected to contribute substantially to heterogeneity within the cohort, as all participants identified as Hispanic/Latino. Importantly, the 2021 CKD-EPI equation used to estimate eGFR does not incorporate a race coefficient. Thus, race-based adjustment was not a source of differential eGFR estimation within this cohort and is unlikely to have introduced bias in comparisons of eGFR decline among participants"
Reviewer requests:
Can this response to reviewer please be incorporated into the article, reporting race and ethnicity (if known) in the table of baseline characteristics?

Response:

Thank you for this helpful suggestion. We have revised the manuscript to explicitly report ethnicity in the description of the study population and included a note about ethnicity in the table of baseline characteristics. "All participants identified as Hispanic/Latino and were recruited from agricultural communities along the Pacific coast of northwestern Nicaragua".

Additionally, we have also clarified in the kidney function analysis subheading that eGFR was estimated using the 2021 CKD-EPI equation, which does not incorporate a race coefficient. Therefore, race-based adjustment was not a source of differential eGFR estimation among participants, reducing the potential for race-related bias in comparisons of kidney-function decline within this cohort.
