# Supplementary material for: Migration, attrition and internal validity in longitudinal study of chronic kidney disease of unknown aetiology in Nicaragua
Source: BMJ Open. 2026 Nov 6;16(11):e122893. doi: 10.1136/bmjopen-2026-122893 (PMC13756283; doi:10.1136/bmjopen-2026-122893)
Supplement: Supplementary file 2 [file bmjopen-16-11-s001.docx]

**Migration, Attrition, and Internal Validity in a Longitudinal Study of Chronic Kidney Disease of Unknown Aetiology in Nicaragua.**

Aurora Aragón^1,2^, Indiana López-Bonilla^1,2^, Neil Pearce^3,4^, Dorothea Nitsch^4^, David Gimeno Ruiz de Porras^5^, Ben Caplin^6^, Marvin González-Quiroz^5,6^

^1^Salud y Trabajo en América Central SALTRA Nicaragua

^2^*Wuqu’ Kawoq Maya Health Alliance, Chimaltenango, Guatemala*

^3^*Department of Medical Statistics, London School of Hygiene and Tropical Medicine, London, UK*

^4^*Department of Non-Communicable Disease Epidemiology, London School of Hygiene and Tropical Medicine, London, UK*

^5^*Department of Environmental and Occupational Health, Kate Marmion School of Public Health, The University of Texas at San Antonio, San Antonio, Texas, USA*

^6^*Centre for Kidney and Bladder Health, University College London, London, UK.*

**Corresponding author:** Aurora Aragón B

*Affiliation:* Salud y Trabajo en América Central SALTRA Nicaragua

*Address:* Residencial Guadalupe, Segunda calle, León, Nicaragua

*Tel:* + (505) 88729722

This **original article** has been seen and approved by all authors listed above and is not under consideration for publication elsewhere.

**Supplementary Tables**

| **Table S1.** Comparison of baseline eGFR <60 ml/min/1.73 m² between participants with and without permanent migration, by cohort and follow-up window. | | | | | |
| --- | --- | --- | --- | --- | --- |
|  |  | **Permanent  migration** | | **95%CI**  **difference in proportions (%)** | **p-value*** |
|  |  | **Yes** | **No** |  |  |
| Over the complete follow-up period | Baseline eGFR < 60 ml/min/1.73 m² | 5 | 16 |  | 1.0000 |
|  | Total | 270 | 731 |  |  |
|  | % with baseline eGFR<60 | 1.9 | 2.2 | - 2.5% to 1.8% |  |
| Cohort 1  (2014) | Baseline eGFR < 60 ml/min/1.73 m² | 2 | 5 |  | 1.0000 |
|  | Total | 104 | 246 |  |  |
|  | % with baseline eGFR<60 | 1.9 | 2.0 | -4.0 to 3.7 |  |
| Cohort 2  (2018) | Baseline eGFR < 60 ml/min/1.73 m² | 3 | 6 |  | 0.7136 |
|  | Total | 117 | 303 |  |  |
|  | % with baseline eGFR<60 | 2.6 | 2.0 | -3.3 to 4.4 |  |
| Cohort 3  (2021) | Baseline eGFR < 60 ml/min/1.73 m² | 0 | 5 |  | 0.5870 |
|  | Total | 49 | 182 |  |  |
|  | % with baseline eGFR<60 | 0.0 | 2.7 | -6.4 to 0.9 |  |
| Within 24 months of follow-up | Baseline eGFR < 60 ml/min/1.73 m² | 3 | 18 |  | 0.7218 |
|  | Total | 112 | 889 |  |  |
|  | % with baseline eGFR<60 | 2.7 | 2.0 | -3.0 to 4.3 |  |
| * P-value from Fisher's exact test used because of sparse cells in some cohorts. | | | | | |

| **Table S2. Reasons for non-migratory loss to follow-up, all cohorts.*** | |
| --- | --- |
| **Reasons** | **n (%)** |
| Work-related absence (left locality for temporary labour but unconfirmed migration) | 58 (75.3%) |
| Haemodialysis (relocated for treatment, did not return) | 1 (1.3%) |
| Caring for parents in Managua | 1 (1.3%) |
| Illness unrelated to CKD | 1 (1.3%) |
| Imprisonment | 1 (1.3%) |
| Death due to CKD | 5 (6.5%) |
| Accidental death | 3 (3.9%) |
| Death due to other medical causes (brain tumour, lung cancer) | 2 (2.6%) |
| Refusal after initial participation | 5 (6.5%) |
| **Total** | 77 (100.0%) |
| * These participants did not meet the definition of permanent migration but were treated as censoring events in time-to-permanent-migration analyses. Deaths were additionally considered as competing events in a sensitivity analysis. | |

| **Table S3.** Sensitivity analysis using complete-case data: multivariable robust regression models of change in eGFR across the three cohorts. (n=949) | | | |
| --- | --- | --- | --- |
| **Characteristics** | **Cohort 1**  **β (95% CI)** | **Cohort 2**  **β (95% CI)** | **Cohort 3**  **β (95% CI)** |
| Permanent migration (Yes) | 2.7 (-4.5 to 10.0) | 2.4 (- 4.4 to 9.3) | 6.3 (-1.1 to 13.7) |
| Sex (Male) | -15.7 (-20.7 to -10.7) | - 20.6 (-25.1 to -16.2) | -15.0 (-19.7 to -10.4) |
| Age (18 to 22) | 0.9 (-4.0 to 5.8) | -2.7 (-6.4 to 1.0) | 1.8 (-3.1 to 6.7) |
| BMI Difference | 0.6 (-0.2 to 1.3) | -0.3 (-0.9 to 0.4) | 0.8 (0.1 to 1.5) |
| Time at Risk (years) | -0.7 (-2.2 to 0.8) | -0.8 (-3.2 to 1.5) | 6.2 (1.7 to 10.6) |
| From multivariate regression models with robust variance. R² values for the 2014, 2018, and 2021 cohorts were 0.090, 0.210, and 0.227, respectively. | | | |

**Figure S1.** Sankey diagram illustrating migration flows stratified by sex and cohort.
